# Early enteral nutrition (EEN) following intestinal anastomosis in pediatric patients – what’s new?

**DOI:** 10.1515/iss-2024-0017

**Published:** 2024-08-20

**Authors:** Sabine Drossard, Louisa Schuffert

**Affiliations:** Department of General, Visceral, Transplant, Vascular and Pediatric Surgery, University Hospital Würzburg, Würzburg, Germany; Department of Pediatric Surgery, University Hospital Mainz, Mainz, Germany

**Keywords:** early enteral nutrition, early oral feeding, intestinal anastomosis, pediatric surgery

## Abstract

**Introduction:**

Abdominal surgery in children may disrupt normal gut function, necessitating prolonged fasting, which can lead to complications such as dehydration and nutritional deficits. Early enteral nutrition (EEN) after surgical procedures can enhance wound healing, prevent malnutrition, and expedite recovery. Although concerns exist regarding the risk of complications associated with EEN, current evidence suggests that it is not linked to increased perioperative complications.

**Content:**

This scoping review provides an overview of the role of EEN in pediatric abdominal surgery, exploring its benefits and risks within the context of recent literature from 2021 to 2024. A systematic literature search was conducted using the PubMed database in April 2024 and the identified studies were compared. The search revealed 586 results, wherefrom eight studies (three systematic reviews and five clinical studies) fulfilled the inclusion criteria. Five studies were added since 2021. Overall, EEN may reduce the length of hospital stay, time to full oral intake, and return of bowel function. It does not seem to increase the rate of anastomotic leakage. EEN is associated with lower rates of surgical site infections and wound dehiscence as well as fewer septic complications. One study showed an increase in nausea/vomiting and abdominal distension in the EEN group, which did not lead to further complications.

**Summary and Outlook:**

Current evidence suggests that EEN after abdominal surgery in pediatric patients is not associated with a higher rate of complications. In fact, EEN seems to be beneficial and lead to improved patient outcomes and shorter hospital stays. Emphasis on patient and parent comfort, individualized feeding initiation based on clinical factors, and standardized postoperative feeding protocols are recommended to optimize outcomes in pediatric abdominal surgery.

## Introduction

In the last decades, there has been growing interest in the benefits of Enhanced Recovery After Surgery (ERAS) protocols and especially Early Enteral Nutrition (EEN) as a core component of ERAS. ERAS aims to optimize postoperative recovery without increasing complication rates. EEN is defined as the initiation of early enteral feeding with varying timeframes from 24 to 72 h after surgery. The ERAS Society consensus guidelines recommend starting enteral nutrition in neonates within 24–48 h after intestinal surgery with human milk as the first choice [1].

Abdominal surgery including bowel anastomosis plays a central role in pediatric surgery, encompassing a spectrum of conditions ranging from malformations or neoplasms to intra-abdominal infections and other diseases that require surgical intervention. Abdominal surgery can impair normal gut function, leading to intestinal paralysis and temporary inability to tolerate oral intake. Fasting may further compromise gut motility and function [2]. Adequate nutrition is essential for optimal healing and recovery after surgery. A well nutritional status can promote wound healing and prevent malnutrition and growth retardation, thus reducing postoperative complications and impacting the length of hospital stay and healthcare costs. EEN may prevent complications such as intestinal paralysis and promote faster recovery of bowel function [1, 2]. Fasting, particularly prolonged fasting in pediatric patients, can lead to a variety of complications like dehydration, electrolyte imbalances, hypoglycemia, and nutritional deficits [2]. These complications can compromise the body’s ability to repair tissues, fight infection, and regain strength, hence negatively impacting perioperative outcomes and prolonging recovery time [1].

Parenteral nutrition (PN) is associated with risks such as metabolic complications and liver dysfunction. The need for central venous access means that there is a higher rate of catheter-associated infections and thrombotic complications in children receiving PN [1]. Therefore, EEN can reduce complications by minimizing the need for PN and central venous access. Enteral nutrition is more physiological and cheaper than parenteral nutrition.

Furthermore, fasting can exacerbate stress, discomfort, and anxiety in pediatric patients, leading to emotional distress in children and their caregivers. EEN in pediatric patients can help alleviate stress and improve the patient’s comfort and well-being as well as their parent’s satisfaction with perioperative management [3], [4], [5].

However, EEN may also have risks, particularly in the situation of intestinal paralysis, when the patient’s gastrointestinal tract is not yet ready to tolerate feeding. In this case, EEN may be associated with an increased risk of aspiration, nausea and vomiting (N/V), or abdominal distension (AD). Furthermore, some surgeons fear increased rates of postoperative complications, notably the incidence of anastomotic leakage (AL) or surgical site infections (SSI) with EEN [2].

The safety and benefits of EEN in adult surgical patients have been established across different surgical disciplines. The European Society of Parenteral Enteral Nutrition recommends commencing enteral nutrition within 24 h of intestinal anastomosis in adult surgical patients [6]. In the field of pediatric surgery, the uptake of EEN following surgical procedures has progressed at a notably slower pace. Traditionally, pediatric surgeons often recommended prolonged postoperative fasting after intestinal anastomosis, believing that “bowel rest” would reduce complication rates and promote anastomotic or ostomy healing [7].

## Research question

In recent years, there has been a surge in interest in the subject of EEN within the pediatric surgery scientific community, leading to an increasing number of studies being conducted. As they cover a diverse range of conditions and surgical interventions, the results may be difficult to interpret. This article aims to provide an overview of existing evidence on the risks and benefits of EEN after gastrointestinal surgery in pediatric patients, focusing on recent literature from the years 2021–2024.

## Methods

A systematic literature search was conducted by using the PubMed database. The search strategy involved the use of controlled vocabulary terms (MeSH terms) and free-text terms. The following search terms were utilized: “early enteral nutrition”, “EEN”, “enhanced recovery after surgery”, “ERAS”, “early enteral feeding”, “early oral feeding”, “fast track” in combination with “pediatric surgery”. To focus on the most recent relevant literature, the search was filtered to only show articles published in 2021–2024.

Articles were included if they were published in peer-reviewed journals, written in English and relevant to the topics of early enteral nutrition after abdominal surgery in pediatric surgery. Original research studies, review articles, guidelines, and other relevant literature types were considered. Articles were excluded if they were not related to the topics of interest, published in languages other than English, or published before the year 2021.

Both authors screened the titles and abstracts of the retrieved articles to identify relevant studies. The full-text articles were then reviewed to determine final inclusion. Discrepancies were resolved through discussion and consensus. Relevant information extracted from each study included authors, publication year, study design, study population, location, outcomes assessed, and key findings. The findings from the included studies were extracted and organized in an Excel spreadsheet (Microsoft Corporation, USA) according to key findings identified across the literature to provide an overview of the current evidence on the role of EEN after intestinal anastomosis in pediatric surgery.

## Results

**Figure 1:**
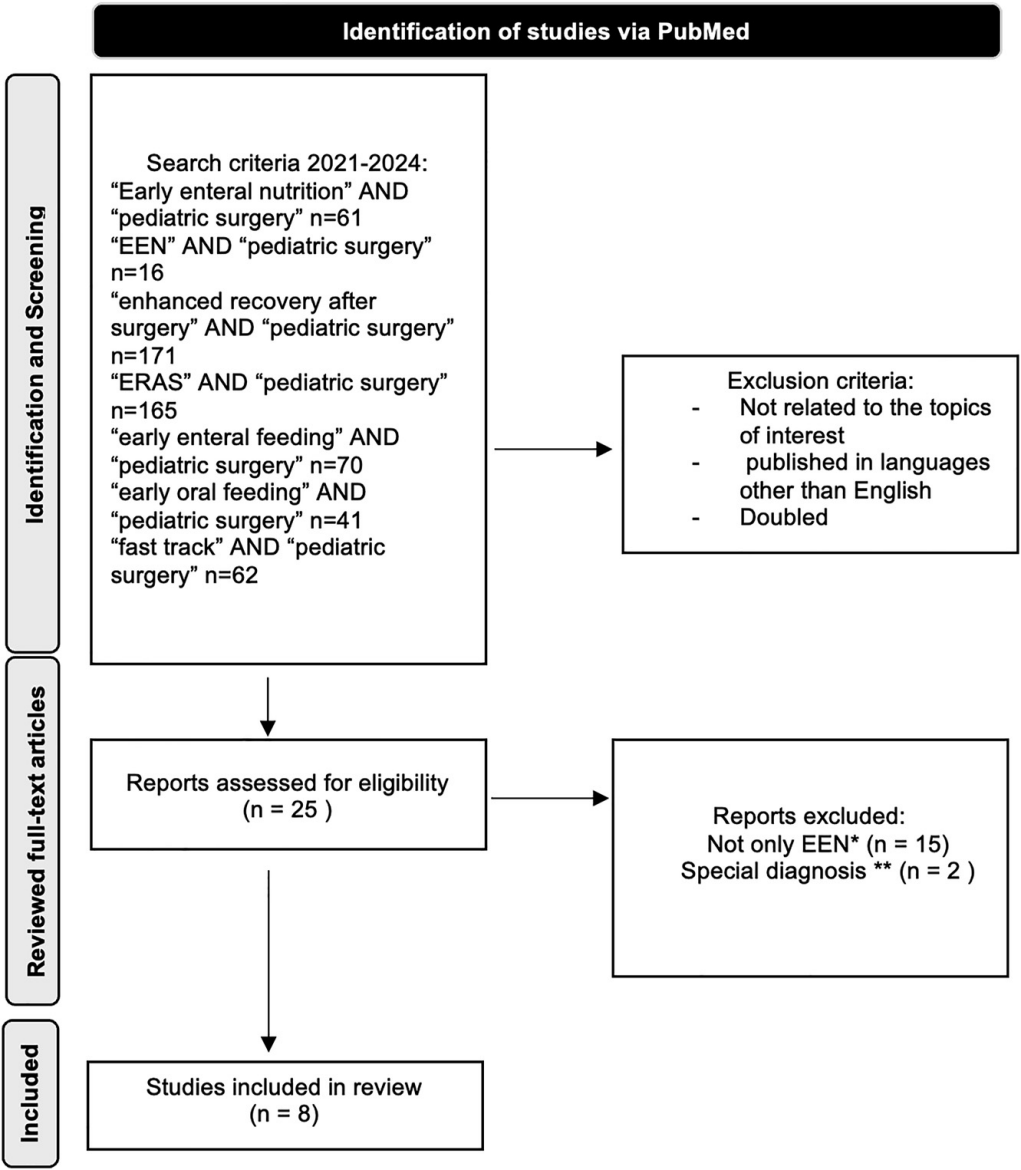
Results of the systematic literature search about the use of EEN in pediatric surgery. *EEN was not the only variable, so there were no conclusions about the effect of EEN measurable. **NEC and gastroschisis, EEN without relation to surgical intervention.

The results of the systematic literature search are depicted in Figure 1 as an adapted PRISMA flow diagram. The search of the 7 keywords in combination with “AND pediatric surgery” from 2021 to 2024 identified 586 search results in PubMed. After screening the articles and their abstracts according to their relation to the topic of interest, 25 articles were identified and reviewed in their full-text version. A total of 17 articles were excluded by applying further exclusion criteria as depicted in Figure 1, leaving eight articles to include.

The summary of study characteristics is shown in Table 1. Of the eight included studies, there were three systematic reviews and meta-analyses [8], [9], [10], one randomized controlled trial [11], two retrospective cohort studies [6, 12], and two observational studies [13, 14]. All studies reported on various types of intestinal anastomosis in pediatric patients. The age groups differ between neonates and children <18 years. The time to first feed in the EEN group varies between <6 and <72 h. All included studies defined length of hospital stay (LOS), seven studies defined abdominal distension (AD) as outcome parameter. Surgical site infection (SSI) and nausea/vomiting (N/V) were defined as outcome parameters by six studies, septic complications and time to stool by five, and wound infections and time to full enteral intake by four studies. Other outcome parameters not investigated in this review are need for and length of PN, malnutrition, albumin and prealbumin levels, duration of analgesia, feeding intolerance, 30-day mortality or readmission rates, and weight for age Z-score (WAZ).

**Table 1:** Studies included in the review.

Authors	Year	Journal	Article type	Country	Intervention	Studies	Sample size		Age group	Time to feeding	Outcome	
							EEN	Control			Primary outcome	Secondary outcome
Issac et al. [9]	12/2023	European Journal of Pediatric Surgery	Systematic review and meta-analysis	–	Gastrointestinal anastomosis in congenital malformations	6	217	271	1 d – 12 y	<48 h	LOS, time to stool, V/N, AL, postoperative wound infection, need for ICU, AD	
Behera et al. [10]	08/2022	Journal of Pediatric Surgery	Systematic review and meta-analysis	–	Intestinal anastomosis	10	614	672	<18 y	< 48 h	AL and other complication (AD, SSI, WD, V, SC, and fever)	Time to stool, LOS
Tian et al. [8]	03/2021	Pediatric Surgery International	Systematic review and meta-analysis of RCTs	–	Intestinal anastomosis	4	97	89	<18 y	< 72 h	AL	SC and fever, V/N, AD, SSI, LOS, time to stool, time to full enteral intake
Cope et al. [13]	04/2024	Journal of Pediatric Surgery	Retrospective cohort study	Australia	Enterostomy closure	–	34	35	3mo – 16 y	< 24 h	LOS	Time to full enteral intake; time to first fluids, time to stool; highest pain score, AD, bowel obstruction, N/V, SSI, AL, WD, SC/fever, 30-day mortality, readmission and reoperation rate
Gil-Vargas et al. [15]	11/2023	Journal of Indian Association of Pediatric Surgeons	Observational study	India	Ileostomy closure		25	20	< 18 y	< 24 h	LOS, time to stool, time to full enteral intake, need of PN, wound infection, SC, malnutrition, average albumin-level	
Jayakumar et al. [12]	10/2023	Journal of Indian Association of Pediatric Surgeons	Retrospective multicentric cohort study	India	Distal bowel anastomosis	–	26	32	< 18 y	< 6 h	Duration of analgesia, LOS, AD, V/N, SSI, readmission	
Lu et al. [14]	07/2023	Frontiers in Nutrition	Prospective, observational multicenter study	China	Intestinal anastomosis	–	182	716	<1 y	<48 h	Time to feeds	V/N, AD, diarrhea, hematochezia, AL, failure of initial FO, PN time, LOS, weight of hospital discharge, albumin, prealbumin at hospital discharge, WAZ
Peng et al. [11]	09/2021	Journal of Pediatric Surgery	Multicenter, prospective, randomized controlled trial	China	Intestinal anastomosis	–	78	78	<28 d	<48 h	LOS, time to full feeds	AD, peritonitis, gastrointestinal hemorrhage, NEC, SC, feeding intolerance, PN duration, feeding intolerance, 30-day mortality and readmission rate, WAZ

PO, primary outcome; SO, secondary outcomes; SSI, surgical site infection; WD, wound dehiscence; AL, anastomotic leakage; SC, septic complications; LOS, length of hospital stay; V/N, vomiting/nausea; AD, abdominal distension; PN, parenteral nutrition.

We included three systematic reviews and five clinical studies. The studies within the systematic reviews were published between 2003 and 2021, whereas the included clinical studies were published in 2021 and later, enabling a comparison of recent findings with older data.

### What is known?

The 2021 meta‐analysis of 10 recent studies with 1,286 patients by Behera et al. concluded that early enteral feeding within 48 h after bowel anastomosis led to earlier discharge as well as fewer surgical site infections and septic episodes [10]. Another meta-analysis published in 2021 by Tian et al. focusing on randomized controlled trials included 186 patients in four studies, showing similar results with no increase in complications and reduction of SSI [8]. In 2023, another systematic review and meta-analysis was added by Issac et al. including 488 patients in six studies and showing similar [9]. The systematic reviews exhibit partial overlap in the included studies (see Table 2) but target different outcomes.

**Table 2:** Overview of studies analyzed in the three included reviews.

Study	Surgical intervention	No. Patients, E/C	Study type	Issac et al. [9]	Behera et al. [10]	Tian et al. [8]
Sangkhataht (Thailand, 2003)	Colostomy closure	64 (34/30)	OS		X	
Ekingen et al. (Turkey, 2005)	Abdominal surgery	56 (33/23)	RCT	X		X
Yadav (India, 2013)	Bowel anastomosis (ileostomy or colostomy closure)	62 (31/31)	OS		X	
Davila-Perez et al. (Mexico, 2013)	Bowel anastomosis	60 (30/30)	RCT	X	X	X
Amanollahi et al. (Iran, 2013)	Resection and anastomosis of small bowel	67 (37/30)	RCT	X	X	X
Paul (Bangladesh, 2015)	Colostomy closure	125 (70/55)	CCT		X	
Shang (China, 2018)	Bowel anastomosis	510 (255/255)	RS		X	
Prasad (India, 2018)	Bowel anastomosis	118 (47/71)	CCT		X	
Ashjaei et al. (Iran, 2019)	Transanal, endorectal pull-through	33 (15/18)	CCT		X	X
Ghosh et al. (India, 2020)	Colostomy closure	147 (45/102)	RCT	X	X	
Iqbal et al. (Pakistan, 2020)	Bowel anastomosis (ileostomy or colostomy closure)	100 (50/50)	RCT	X	X	
Khademi et al. (Iran, 2021)	Esophageal repair	58 (25/23)	RCT	X		

E, experimental group; C, control group; OS, observational study; RCT, randomized controlled trial; CCT, controlled clinical trial; RS, retrospective study.

### What is new?

Only one multicenter, prospective, randomized controlled trial was added in 2021 by Peng et al. on 156 neonates undergoing surgery for congenital gastrointestinal malformation. Even though there were no significant differences for the primary outcomes of time to full feeds or length of hospital stay, the study did demonstrate the safety of initiating early feeding as there were no significant changes in complication rates. Furthermore, postoperative outcomes demonstrated a trend toward improvement that was not statistically significant [11]. A retrospective cohort study (“EPOC”) examining 69 children showed that EEN is efficacious and safe for children undergoing intestinal anastomosis following stoma closure. It was associated with shorter length of stay, time to free fluids, and time to full enteral intake, without increasing complication rates. Another retrospective cohort study conducted in India examined 58 children and showed that EEN after elective intestinal anastomosis is safe and associated with a significant reduction in analgesic needs [12]. The observational study by Lu et al. conducted in China analyzed 898 children, showing a reduction in PN usage without increase of complication rates [13].

The results of this review are shown in Table 3: EEN reduces length of hospital stay. It seems to reduce time to full oral intake and return of bowel function. It does not increase the rate of anastomotic leakage. EEN is associated with lower rates of surgical site infections and wound dehiscence as well as fewer septic complications. One study showed an increase in nausea/vomiting and abdominal distension in the EEN group, which did not lead to further complications.

**Table 3:** Outcomes of the studies included in the review.

Article	SSI	WD	AL	N/V	AD	SC	FEI	RBO	LOS
Cope et al. [13]	⇔	⇔	⇔	✕	✕	⇔	**⇩**	**⇩**	**⇩**
Gil-Vargas et al. [15]	⇔	**⇩**	✕	✕	⇔	**⇩**	**⇩**	**⇩**	**⇩**
Jayakumar et al. [12]	**⇩**	✕	⇔	**⇩**	**⇩**	✕	✕	✕	**⇩**
Lu et al. [14]	✕	✕	⇔	**⇧**	**⇧**	✕	✕	✕	**⇩**
Peng et al. [11]	✕	✕	⇔	⇔	⇔	⇔	⇔	✕	⇔

**Reviews**									

Issac et al. [9]	**⇩**	**⇩**	⇔	⇔	**⇩**	✕	✕	**⇩**	**⇩**
Behera et al. [10]	**⇩**	⇔	⇔	⇔	⇔	**⇩**	✕	**⇩**	**⇩**
Tian et al. [8]	**⇩**	✕	⇔	⇔	⇔	⇔	**⇩**	**⇩**	**⇩**

SSI, surgical site infection; WD, wound dehiscence; AL, anastomotic leakage; V/N, vomiting/nausea; AD, abdominal distension; SC, septic complications; FEI, time to full enteral intake; RBO, time to return of bowel function; LOS, length of hospital stay; X, no data; double-headed arrow, no change; arrow up, increase; arrow down, decrease.

## Discussion

The recently added clinical studies confirm that EEN decreases length of hospital stay and suggest that it decreases time to full enteral intake and return of bowel function. They also indicate that there are no increased rates of anastomotic leakage or septic complications. Unlike the systematic reviews, only one clinical study showed a significant decrease in surgical site infections, while the others showed no significant difference. These findings are consistent with the observations reported in a prior review [7].

Even though the available research is of low to medium quality, there is no evidence that EEN leads to a higher rate of perioperative complications after intestinal anastomosis in pediatric patients. Thus, the traditional approach of waiting on a bowel movement to start feeds is not backed up by evidence. On the contrary, current evidence suggests that EEN may in fact be associated with better postoperative outcomes.

In this review, we focused on the effect of EEN as only one, but a core tenant of ERAS. However, there are several studies on ERAS in pediatric surgery that are in line with the findings of this review: a study on pediatric patients undergoing colostomy reversal showed shorter length of hospital stay, fewer surgical site infections, and lower overall complication rates in the ERAS group with feeding starting on the first postoperative day [15]. A meta-analyses of 1,153 pediatric urology patients described earlier enteral feeding and return of bowel function in the ERAS groups with no increase in complication rates [16]. Another study showed that EEN is safe after primary repair of anorectal malformations without protective stoma as there was no higher risk for wound infection or dehiscence in the EEN group [17].

There are several explanations why EEN may be associated with better postoperative outcomes. By now, several animal studies have shown that collagen content in anastomotic tissues is reduced in prolonged fasting, thus impairing healing. Early enteral nutrition on the other hand has been shown to increase collagen deposition and, therefore, strength of bowel anastomosis [13]. Fasting leads to atrophy of the villi. Decreased activity of intestinal enzymes and channels modifies the permeability of the intestinal mucosa to antigens and macromolecules. The gut flora changes and with the absence of a normal flora, colonization of pathological microorganism can begin and may lead to sepsis [14]. Optimization of nutritional status in patients receiving EEN may be another explanation: malnutrition can delay postoperative recovery by compromising the body’s metabolism, which can have a negative impact in wound healing and recovery.

Furthermore, patient and parent comfort are crucial considerations in pediatric surgery. Prioritizing patient well-being may also play a significant role in optimizing postoperative care: comfortable children experience less anxiety and are more likely to cooperate with medical interventions. Likewise, satisfied parents are more likely to trust the medical team and adhere to postoperative care instructions. Both aspects might contribute to smoother recovery and improved patient outcomes. Regrettably, none of the studies incorporated in the analysis quantified the satisfaction levels of either the patients or their guardians. Notably, a study conducted by Zhang et al. demonstrated that adherence to an ERAS protocol, inclusive of EEN, resulted in significantly augmented parental satisfaction when compared to the control group [12].

## Limitations

In this scoping review, we incorporated three systematic reviews that exhibit partial overlap in the studies they encompass. This overlap could potentially give an exaggerated perception of the robustness of the evidence. Furthermore, all included studies of the systematic reviews predate 2021. These reviews were selected for inclusion to facilitate comparison with more recent clinical investigations.

Although some new research was added to the topic, the quality of the studies is still low. Most studies are designed as retrospective cohort or observational studies. Only one prospective randomized controlled trial was added in 2021. The included studies exhibit high heterogeneity in terms of age variance, reasons for surgery, feeding protocols, and other variants. Consequently, only tentative statements can be made based on these findings.

Larger randomized controlled trials are required to further assess the benefits of EEN and its impact on postoperative complications in pediatric surgery. Furthermore, the effect on patient- and parent-reported outcome measures and satisfaction should be investigated.

### Limitations to the implementation of EEN protocols

Surgery and pediatric surgery have long displayed a lack of evidence-based decision-making. Often perioperative management relies on clinical experience and surgeon preference [18].

EEN is only one aspect of ERAS, which includes several aspects of pre-, peri-, and postoperative handling. Therefore, ERAS protocols can only be implemented in a holistic, interdisciplinary approach. It has been recommended to allow the patient to guide oral intake by relying on signs of hunger and factors like abdominal distension, nausea or vomiting [1]. A survey among pediatric surgeons about the utilization of ERAS protocols in minimal invasive surgery described “existing culture” and “lack of education” as major barriers to their implementation [19]. Pediatric surgical patients are often cared for by pediatric or neonatal intensive care teams with surgeons more or less closely involved in postoperative care and nutrition decisions. This leads to challenges when implementing new postoperative protocols. Close interdisciplinary collaboration is vital to ensure optimal nutritional support and promote favorable outcomes in this patient population. In other settings like pediatric cardiac surgery or infants with gastroschisis, it has been shown that the implementation of standardized postoperative feeding protocols resulted in earlier feedings and reach of feeding goals. Benefits may be realized just by standardizing postoperative feeding through simplifying the approach for healthcare providers and families [1]. Therefore, the development and implementation of standardized postoperative feeding protocols should be fostered. Close monitoring and collaboration between surgical teams, pediatric and intensive care teams, and other healthcare providers are essential to optimize nutritional support and promote the best outcomes for pediatric patients undergoing abdominal surgery.

## Conclusions

Current evidence suggests that early enteral nutrition after abdominal surgery in pediatric patients is not associated with a higher rate of complications. In fact, EEN seems to be beneficial and lead to improved patient outcomes and shorter hospital stays.

Patient and parent comfort are crucial considerations in pediatric surgery. Avoiding unnecessary fasting can help alleviate anxiety and promote emotional well-being during the perioperative period in pediatric patients, hence contributing to improved outcomes and overall satisfaction in pediatric surgical patients and their families. The timing of enteral nutrition initiation is an individualized decision based on the patient’s clinical condition, surgical procedure, gastrointestinal function, and tolerance to feeding. Enteral nutrition should be prioritized whenever feasible due to its critical role in maintaining gut function, preserving the integrity of the gut mucosa and preventing malnutrition and dehydration.
